# Structural abnormalities in cortical volume, thickness, and surface area in 22q11.2 microdeletion syndrome: Relationship with psychotic symptoms^☆^

**DOI:** 10.1016/j.nicl.2013.09.013

**Published:** 2013-10-14

**Authors:** Maria Jalbrzikowski, Rachel Jonas, Damla Senturk, Arati Patel, Carolyn Chow, Michael F. Green, Carrie E. Bearden

**Affiliations:** a Department of Psychology, University of California, 1285 Franz Hall, Box 951563, Los Angeles, CA 90095-1563, USA; b Department of Psychiatry and Biobehavioral Sciences, Semel Institute for Neuroscience and Human Behavior, University of California, 760 Westwood Plaza, Los Angeles, CA 90095, USA; c Department of Biostatistics, School of Public Health, University of California, Los Angeles, CA 90095, USA; d VA Greater Los Angeles Healthcare System, VISN22 Mental Illness Research, Education and Clinical Center, 11301 Wilshire Blvd., Los Angeles, CA 90073, USA

**Keywords:** 22q11DS, 22q11.2 deletion syndrome, CNV, copy number variation, CT, cortical thickness, SA, surface area, SIPS, Structured Interview for Prodromal Syndromes, MRI, magnetic resonance imaging, ANCOVA, analysis of covariance, Copy number variation, Structural magnetic resonance imaging, Psychosis, Schizophrenia, Velocardiofacial syndrome

## Abstract

**Introduction:**

22q11.2 deletion syndrome (22q11DS) represents one of the largest known genetic risk factors for psychosis, yet the neurobiological mechanisms underlying symptom development are not well understood. Here we conducted a cross-sectional study of 22q11DS to decompose cortical volume into its constituent parts, cortical thickness (CT) and surface area (SA), which are believed to have distinct neurodevelopmental origins.

**Methods:**

High-resolution T1-weighted scans were collected on 65 participants (31 22q11DS, 34 demographically comparable typically developing controls, 10–25 years old). Measures of cortical volume, CT, and SA were extracted from regions of interest using the FreeSurfer image analysis suite. Group differences and age-related trajectories in these structures, as well as their association with psychotic symptomatology, were assessed.

**Results:**

Relative to controls, 22q11DS participants showed bilateral volumetric reductions in the inferior temporal cortex, fusiform gyrus, anterior cingulate, superior parietal cortex, and cuneus, which were driven by decreased SA in these regions. 22q11DS participants also had increased volumes, driven by increased CT, in bilateral insula regions. 22q11DS youth had increased CT in frontal regions, particularly middle frontal and medial orbitofrontal cortices. A pattern of age-associated cortical thinning was observed in typically developing controls in brain regions associated with visual and sensory information-processing (i.e., left pericalcarine cortex and fusiform gyrus, right lingual and postcentral cortices). However, this relationship was disrupted in 22q11DS participants. Finally, correlational analyses revealed that increased CT in right medial orbitofrontal cortex was associated with increased positive symptom severity in 22q11DS.

**Conclusion:**

Differential disruptions of CT and SA in distinct cortical regions in 22q11DS may indicate abnormalities in distinct developmental neural processes. Further, neuroanatomic abnormalities in medial frontal brain structures disproportionately affected in idiopathic schizophrenia were associated with psychotic symptom severity in 22q11DS youth, suggesting that disrupted biological processes in these cortical regions may underlie development of psychotic symptoms, both in 22q11DS and in the broader population.

## Highlights

• 22q11DS offers a valuable model for neurobiological mechanisms of psychosis.
• First study of multiple structural MRI indices in 22q11DS.
• Reduced surface area in multiple neuroanatomic regions in 22q11DS.
• Increased cortical thickness in multiple frontal regions and insula in 22q11DS.
• Orbitofrontal abnormalities associated with positive symptom severity in 22q11DS.

## 1 Introduction

Recent evidence regarding the genetic architecture of schizophrenia suggests that rare genetic mutations, known as copy number variations (CNVs), may play a larger role in the disorder's etiology than was previously believed. One of the best-known CNVs associated with schizophrenia is the 22q11.2 microdeletion syndrome (22q11DS). Individuals with 22q11DS have substantially elevated rates of psychotic illness, as high as 32% (Gothelf et al., 2007a), making it one of the largest known risk factors for developing schizophrenia. 22q11.2 deletions account for about 1–2% of schizophrenia cases in the general population (Bassett et al., 2010). Moreover, the core clinical phenotype in schizophrenia patients with 22q11DS does not differ from schizophrenia patients without the deletion (Bassett et al., 2003; Murphy et al., 1999). This syndrome therefore represents an excellent model for investigating how a known genetic mutation may lead to structural brain dysfunction, which in turn may relate to expression of psychotic symptoms.

Meta-analyses of volumetric and voxel-based structural magnetic resonance imaging studies of patients with idiopathic schizophrenia have found consistent volumetric reductions in frontal (Fornito et al., 2009; Hulshoff Pol and Kahn, 2008; Shepherd et al., 2012), temporal (Honea et al., 2005; Hulshoff Pol and Kahn, 2008; Shepherd et al., 2012), and medial temporal regions (Glahn et al., 2008), along with the insula (Ellison-Wright et al., 2008; Glahn et al., 2008) and anterior cingulate (Ellison-Wright et al., 2008; Glahn et al., 2008; Shepherd et al., 2012). A recent study used surface-based methodology to distinguish between cortical thinning and reduced cortical surface area in a large sample of patients with schizophrenia, revealing that volumetric reductions in these regions are primarily driven by reduction in cortical thickness (CT), although reduced surface area (SA) was also present in more circumscribed regions (Rimol et al., 2012).

There is some evidence that neuroanatomic regions typically disrupted in idiopathic schizophrenia are also linked to psychotic symptomatology in 22q11DS. Adults with 22q11DS and a schizophrenia diagnosis have reduced frontal and superior temporal gyrus volumes, in comparison to those with 22q11DS without a schizophrenia diagnosis (Chow et al., 2002, 2011). In 22q11DS youth, volumetric reductions in the bilateral cingulate gyrus (Dufour et al., 2008) and reduced temporal gray matter have been associated with psychotic symptoms and severity of thought problems (e.g., hearing things, having strange beliefs, displaying odd behaviors), as measured by the Child Behavior Checklist (Bearden et al., 2004), respectively. Also, decreases in superior temporal gyrus volume predicted positive symptoms at a 3-year follow-up in adolescents with 22q11DS (Kates et al., 2011a). Collectively, these findings suggest that neuroanatomic alterations in fronto-temporal brain regions may underlie psychotic symptom development in 22q11DS.

However, no studies have yet investigated the independent contributions of CT and SA to volumetric differences in 22q11DS. Recent evidence suggests that these indices may be driven by different genetic and neurobiological mechanisms. Two studies found that both indices are heritable, yet they are not genetically correlated with each other, indicating that measures of cortical volume combine at least two distinct sources of genetic effects (Winkler et al., 2010). SA and CT are also believed to arise from different progenitor cells, which create glial cells and neurons at distinct points in corticogenesis, resulting in SA ultimately reflecting the number of cortical columns and CT representing numbers of neurons within the columns (Pontious et al., 2008; Rakic, 1988). Thus, identifying differential contributions of these sub-components of cortical volume in 22q11DS may help us better understand abnormal developmental processes that occur during corticogenesis in distinct brain regions in this syndrome.

This is a cross-sectional study examining alterations in cortical volume, thickness, and SA in individuals with 22q11DS as compared to demographically comparable controls. Based on previous findings, we hypothesized that those with 22q11DS would have increased volume in the insula (Simon et al., 2005), with concomitant volumetric reductions in parieto-occipital (Bearden et al., 2007; Gothelf et al., 2007b; Kates et al., 2001), temporal (Campbell et al., 2006; Chow et al., 2002; Eliez et al., 2001), orbitofrontal (Kates et al., 2011b), and anterior cingulate cortices (Dufour et al., 2008). Secondly, we wished to investigate whether there was an altered age-associated trajectory of structural changes in these brain regions in 22q11DS, given the important role of adolescent neurodevelopmental changes in the onset of psychosis (Paus et al., 2008). We hypothesized that the typical adolescent developmental trajectory of cortical thinning with increasing age (Fjell et al., 2009; Tamnes et al., 2010) would be exaggerated in those with 22q11DS, considered that increased cortical thinning has been shown in idiopathic schizophrenia (Cobia et al., 2012) and individuals who go on to develop psychosis (Jung et al., 2011; Sun et al., 2009). Finally, we investigated whether neuroanatomic variation in neuroanatomic regions, as indexed by CT and SA, accounts for variability in positive symptoms in 22q11DS patients.

## 2 Methods

### 2.1 Participants

The total sample consisted of 65 participants (10–25 years old, 31 22q11DS and 34 controls), recruited from an ongoing longitudinal study at the University of California, Los Angeles (UCLA). 22q11DS participants all had a molecularly confirmed diagnosis of 22q11.2 deletion syndrome. Exclusion criteria for all study participants were: neurological or medical condition disorder that might affect performance, insufficient fluency in English, and/or substance or alcohol abuse and/or dependence within the past 6 months. Controls additionally must not meet criteria for any major mental disorder, with the exception of attention deficit-hyperactivity disorder (ADHD) or past episode of depression, based on information gathered during administration of the Structured Clinical Interview for DSM-IV Axis I Disorders (First et al., 1997).

After study procedures had been fully explained, and participants under the age of 18 years provided written assent, while their parent or guardian completed written consent. The UCLA Institutional Review Board (IRB) approved all study procedures and informed consent documents.

### 2.2 Structured Interview for Prodromal Syndromes

A master's-level trained clinician assessed all participants on the positive, negative, disorganized, and general symptom scales from the Structured Interview for Prodromal Syndromes (SIPS; McGlashan, 2001). Symptoms on these scales are rated from 0 to 6, with zero representing an absence of symptoms and six referring to an extremely severe level of symptoms. All raters demonstrated good to excellent inter-reliability for symptom ratings, with intra-class correlation coefficients (ICC's) ranging from 0.85 to 1.00. For the purposes of this study, SIPS positive symptom total score was used as a dimensional measure of psychotic symptoms (i.e., unusual thought content, suspiciousness, grandiose ideas, perceptual abnormalities, and/or disorganization communication). This measure encompasses a range of symptom severity, including sub-threshold (prodromal) and fully psychotic symptoms. Use of a dimensional measure that captures sub-threshold psychotic symptoms (i.e., the SIPS) is important, given that our age range falls within an “at-risk” developmental period.

### 2.3 MRI acquisition

All scanning were carried out on a Siemens 3 T “Tim Trio” MRI scanner at the Brain Mapping Center at UCLA (22q11DS = 17, controls = 17) or at the Center for Cognitive Neuroscience (22q11DS = 14, controls = 17). Measures of brain structure were obtained with high-resolution structural MRI. Each scan began with a 10-min acquisition of standard images used for determining regional anatomy, including a sagittal localizer image (TR/TE = 500/33 ms, 192 × 256 matrix), a high-resolution T2-weighted axial image (TR/TE = 5000/33 ms, 128 × 128 matrix, FOV = 200 × 200mm), and a sagittal 1 mm^3^ T1-weighted image (MPRAGE, TR/TE = 2300/2.91 ms, flip angle = 9°; slice thickness = 1.20 mm, 240 × 256 acquisition matrix).

### 2.4 MRI analysis

The FreeSurfer image analysis suite (version 5.0, http://surfer.nmr.mgh.harvard.edu) surface-based processing pipeline was used to derive measures of volume, CT, and SA. FreeSurfer is a well-validated processing protocol that has been previously described in detail (Dale et al., 1999; Fischl et al., 1999). In short, the following steps were taken in the processing stream: motion correction, transformation of images to standard Talairach space, intensity normalization, removal of non-brain tissue, segmentation of white matter and subcortical structures, and final segmentation of cortical surfaces. Final segmentation is based on both a subject-independent probabilistic atlas and subject-specific measured values. Raters (MJ, AP, RJ) blind to diagnosis visually inspected the scans at several points along the processing pipeline and any errors were manually edited. Details regarding the manual editing procedures can be found in the Supplementary text. Using an automated computer algorithm, CT estimates were derived by taking the distance between the gray–white matter border and the pial surface at each vertex (Fischl and Dale, 2000). As in prior publications (Bhojraj et al., 2011; Gutierrez-Galve et al., 2010; Meda et al., 2012; Venkatasubramanian et al., 2012), surface area was calculated by taking the sum of the area of the vertices in each parcellation. Volume was then calculated as the product of the SA and CT for each region.

The FreeSurfer image analysis suite (version 5.0, http://surfer.nmr.mgh.harvard.edu) surface-based processing pipeline was used to derive measures of volume, CT, and SA. FreeSurfer is a well-validated processing protocol that has been previously described in detail (Dale et al., 1999; Fischl et al., 1999). In short, the following steps were taken in the processing stream: motion correction, transformation of images to standard Talairach space, intensity normalization, removal of non-brain tissue, segmentation of white matter and subcortical structures, and final segmentation of cortical surfaces. Final segmentation is based on both a subject-independent probabilistic atlas and subject-specific measured values. Raters (MJ, AP, RJ) blind to diagnosis visually inspected the scans at several points along the processing pipeline and any errors were manually edited. Details regarding the manual editing procedures can be found in the Supplementary text. Using an automated computer algorithm, CT estimates were derived by taking the distance between the gray–white matter border and the pial surface at each vertex (Fischl and Dale, 2000). As in prior publications (Bhojraj et al., 2011; Gutierrez-Galve et al., 2010; Meda et al., 2012; Venkatasubramanian et al., 2012), surface area was calculated by taking the sum of the area of the vertices in each parcellation. Volume was then calculated as the product of the SA and CT for each region.

**Fig. 1:**
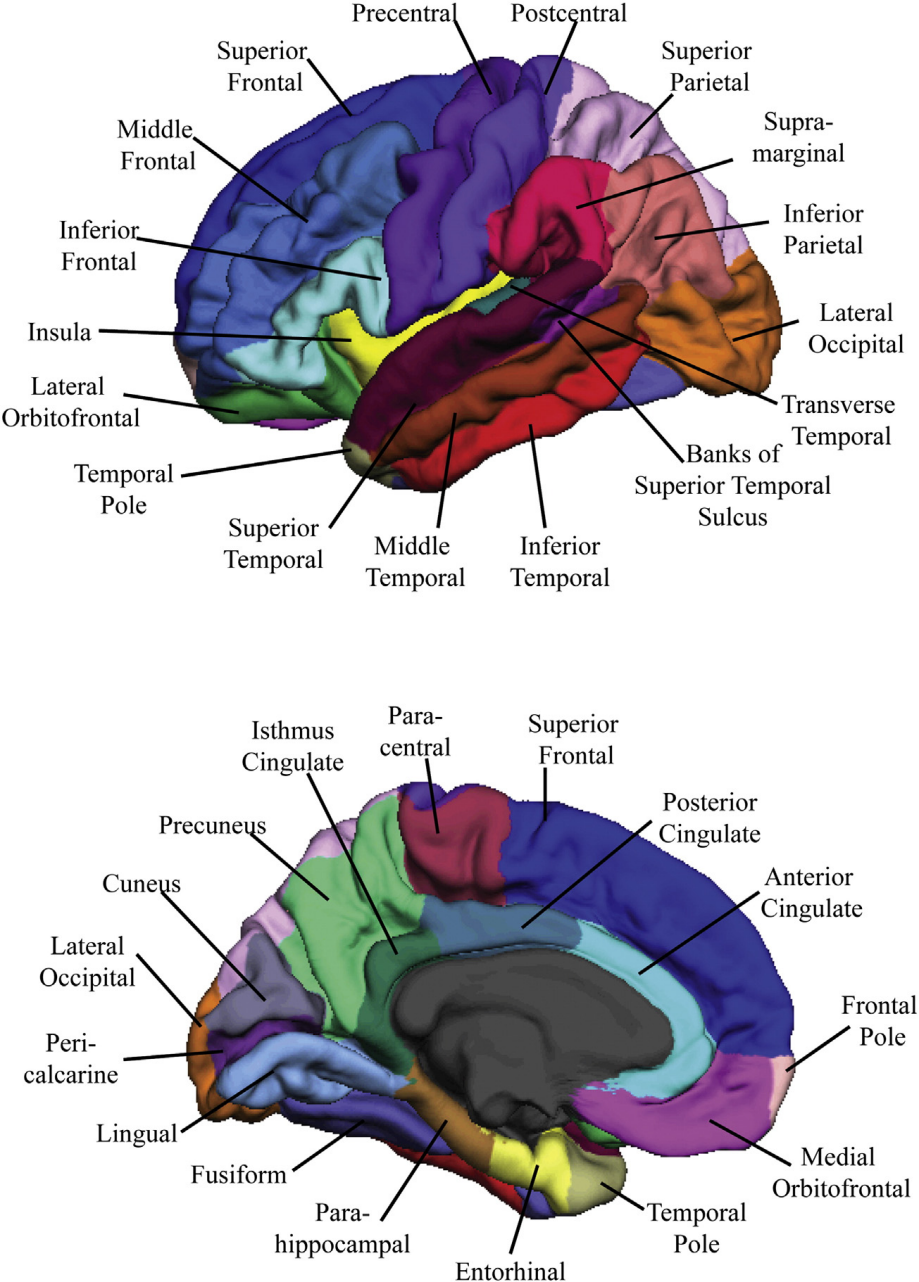
Neuroanatomic regions of interest examined in 22q11DS vs. typically developing controls, in lateral (left) and medial (right) cortical structures.

We extracted values based on the Desikan FreeSurfer atlas (Desikan et al., 2006). For two of the regions of interest, the middle frontal cortex and anterior cingulate, the Desikan atlas (Desikan et al., 2006) separately calculates caudal and rostral regions. Prior to extracting the data, we used the merge label function in FreeSurfer to combine the caudal and rostral labels for each region (i.e., caudal and rostral anterior cingulate labels were merged together to make an anterior cingulate label, caudal and rostral middle frontal labels were merged together to make a middle frontal cortex label). Additionally, we used this approach to combine the pars orbitalis, pars triangularis, and pars opercularis to create the inferior frontal cortex ROI. Then we extracted cortical volume, SA, and CT from these regions, resulting in a total of 30 regions per hemisphere (Fig. 1).

### 2.5 Statistical analyses

Statistical analyses were performed using SPSS software v. 21 (IBM, Chicago, IL) and SAS/STAT software (SAS Institute Inc., Cary, NC, USA). We compared demographic characteristics between groups using independent samples t-tests for continuous variables and chi-square test for categorical variables. To ensure that there were no cross-scanner differences, for all neuroanatomic measurements, we conducted a univariate analysis of covariance (ANCOVA) for each identified region in each hemisphere, with scanner location as the between-group factor and group as a covariate.

All neuroanatomic measures were examined for normality using the Kolmogorov–Smirnov and Shapiro–Wilk tests and transformed appropriately if they violated assumptions of normality. To compare global measures of total intracranial volume, total cortical volume, SA, and CT between 22q11DS participants and controls, we conducted a univariate ANCOVA with each neuroanatomic measure as the dependent variable, diagnosis as the between-group factor, and age, gender, and scanner location as covariates. To compare brain volume, CT, and SA in 22q11DS vs. controls, we conducted an ANCOVA for each identified region in each hemisphere, with diagnosis (22q11DS vs. control) as the between subject factor and total intracranial brain volume/SA, sex, scanner site, and age as the covariates. To ensure that outliers were not driving any of our significant differences, these analyses were also conducted with outliers (those with values > 3 standard deviations away from the mean value) removed.

To address whether the relationship between age and neuroanatomic measures differed between groups, we first visually examined the scatter plots. In plots that visually indicated age ∗ group interactions, we then added age ∗ group interaction terms to the original ANCOVA models for each brain region. This resulted in a total of 14 analyses. We examined volume in the left paracentral cortex and right precuneus, CT in the right frontal pole, lateral occipital and lingual cortices and left pericalcarine region, and in bilateral middle frontal, precuneus, postcentral and fusiform cortices. If significant group ∗ age interactions were detected, Pearson correlations between the ROI and age were calculated for each group (22q11DS and controls) separately. Then, to directly compare the strength of correlations between the two groups, a Fisher r-to-z transformation was conducted.

For statistical analyses corresponding to the third aim of our study (i.e., to determine whether the brain regions investigated above are associated with variability in positive symptoms in 22q11DS patients), we examined the relationship between CT and SA and SIPS positive symptom scores in 22q11DS. Because cortical volume is derived from these two indices, we did not additionally examine volume in relation to positive symptoms. Residuals were calculated from each variable, after regressing out the effects of age and sex. Then, Pearson correlations (corresponding to age- and sex-adjusted partial correlations) were conducted between each brain region with residualized positive symptoms.

False discovery rate (FDR) *q*-values were used to correct for multiple comparisons (300 comparisons in total), and were estimated using SAS/STAT software (SAS Institute Inc., Cary, NC, USA), with a threshold of *q* < .05 considered statistically significant. We also note statistical trends in the results (*q*-values > .05 and < .20), as potentially interesting for investigation in future, larger-scale studies.

## 3 Results

As shown in Table 1, 22q11DS patient and control groups were matched on all demographic factors (all *p*-values ≥ 0.10). There were no between-scanner differences in volume, CT, or SA that survived multiple comparison correction (see Supplemental materials, Table 1). Nevertheless, because some statistical trends emerged for specific structures we covaried for scanner site in all subsequent analyses. Statistics for global measures of intracranial volume, whole brain cortical volume, CT, and SA are reported in the Supplementary materials, Table 2. In comparison to controls, 22q11DS participants showed significantly decreased total intracranial volume, whole brain volume, and total SA, but increased overall mean CT.

As shown in Table 1, 22q11DS patient and control groups were matched on all demographic factors (all *p*-values ≥ 0.10). There were no between-scanner differences in volume, CT, or SA that survived multiple comparison correction (see Supplemental materials, Table 1). Nevertheless, because some statistical trends emerged for specific structures we covaried for scanner site in all subsequent analyses. Statistics for global measures of intracranial volume, whole brain cortical volume, CT, and SA are reported in the Supplementary materials, Table 2. In comparison to controls, 22q11DS participants showed significantly decreased total intracranial volume, whole brain volume, and total SA, but increased overall mean CT.

### 3.1 Volumetric results

Results of group comparisons for brain volumes are presented in Table 2. After correcting for multiple comparisons, individuals with 22q11DS had significantly smaller volumes than controls in the anterior cingulate, fusiform gyrus, cuneus, precuneus, superior parietal and inferior temporal cortices bilaterally, and in the right precentral and middle frontal cortices. In contrast, in bilateral regions of the insula and the right medial orbitofrontal cortex, individuals with 22q11DS had greater volumes than controls. The greatest effect sizes were observed for the cuneus (left: partial *η^2^* = .40; right: partial *η^2^* = .25), right insula (partial *η^2^* = .27) and left inferior temporal cortex (partial *η^2^* = .28).

### 3.2 Surface area results

**Fig. 2:**
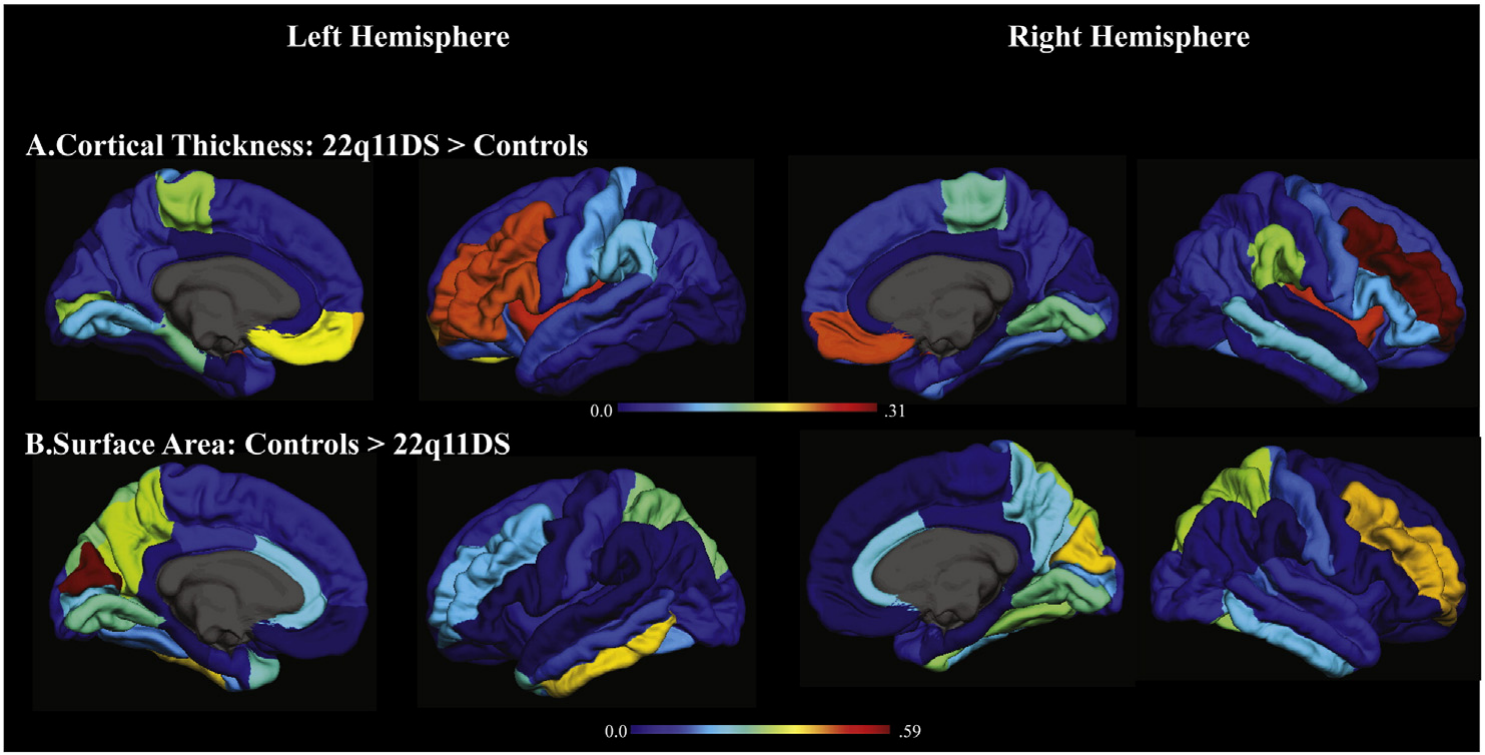
Increases in cortical thickness (A) and decreases in surface area (B) in 22q11DS vs. typically developing controls. Shows an overlay of effect size (partial *η^2^*, values indicated by the color bar) on each FreeSurfer region of interest between those with 22q11DS and typically developing controls. Warm colors indicate a larger effect size, or a greater difference between 22q11DS vs. controls.

Results for the group comparisons of brain SA are also presented in Table 2. F-value overlays are presented in Fig. 2. In comparison to controls, 22q11DS participants had significantly reduced SA in bilateral anterior cingulate, cuneus, precuneus, fusiform gyrus, lingual, pericalcarine, superior parietal, middle frontal, and inferior temporal cortices, as well as in right banks of the superior temporal sulcus, right superior temporal, right postcentral, left temporal pole and left middle temporal regions, in comparison to controls. The greatest effect sizes were observed for the cuneus (left: partial *η^2^* = .59; right: partial *η^2^* = .40), right middle frontal (partial *η^2^* = .41), left inferior temporal (partial *η^2^* = .39), and right superior parietal regions (partial *η^2^* = .32).

### 3.3 Cortical thickness results

A different pattern of results emerged when examining group differences in CT (see Table 2, Fig. 2). In comparison to controls, individuals with 22q11DS had increased CT in bilateral regions of the insula, paracentral, middle frontal, and medial orbitofrontal cortices, as well as right middle temporal, lingual, and supramarginal regions, and the left frontal pole, inferior frontal, pericalcarine cortices. The greatest effect sizes were observed for the middle frontal cortices (left partial *η^2^* = .24; right: partial *η^2^* = .31), medial orbitofrontal regions (left partial *η^2^* = .19; right: partial *η^2^* = .24), and insula (left partial *η^2^* = .24; right: partial *η^2^* = .31). In contrast, 22q11DS participants had decreased CT in the left parahippocampal region, relative to controls (partial *η^2^* = .14).

### 3.4 Age ∗ group interactions

**Fig. 3:**
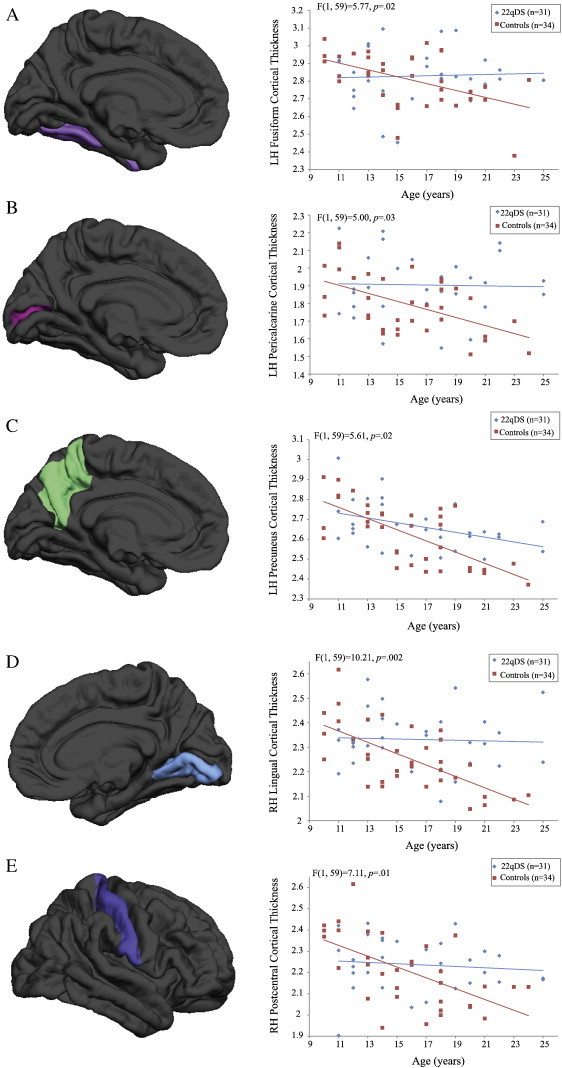
Distinct patterns of age-associated changes in cortical thickness in 22q11DS patients relative to typically developing controls in the (A) left fusiform gyrus, (B) left pericalcarine cortex, (C) left precuneus, (D) right lingual cortex, and (E) right postcentral cortex. In each region, controls show the expected pattern of cortical thinning with increasing age, which is not observed in 22q11DS patients. F-statistic is reported for each significant interaction.

For the fourteen measurements in which visual inspection suggested an age ∗ group interaction, interaction terms were added to the original ANCOVAs examining group differences. Specifically, significant age ∗ group interactions were observed for CT in multiple regions (see Fig. 3): the left pericalcarine cortex (F(1,59) = 5.00, *p* = .03, partial *η^2^* = .08), fusiform gyrus (F(1,59) = 5.77, *p* = .02, partial *η^2^* = .09), precuneus (F(1,59) = 5.61, *p* = .02, partial *η^2^* = .09), as well as the right lingual (F(1,59) = 10.21, *p* = .002, partial *η^2^* = .15) and postcentral cortices (F(1,59) = 7.11, *p* = .01, partial *η^2^* = .11). Age ∗ group interaction terms did not reach statistical significance for the following regions: volumetric measures of left paracentral cortex and right precuneus, CT in the right frontal pole, fusiform gyrus, precuneus, and lateral occipital cortex, left postcentral cortex, and in bilateral middle frontal regions. For all regions that showed a significant age ∗ group interaction, controls showed a highly significant pattern of decreasing CT with increasing age, which was not observed in 22q11DS participants. Fisher's test for the equivalence of correlations showed that, in four of these regions (left pericalcarine and fusiform regions, right lingual and postcentral cortices), the strength of the correlation was significantly greater in controls relative to 22qDS participants (Supplementary Table 3).

For the fourteen measurements in which visual inspection suggested an age ∗ group interaction, interaction terms were added to the original ANCOVAs examining group differences. Specifically, significant age ∗ group interactions were observed for CT in multiple regions (see Fig. 3): the left pericalcarine cortex (F(1,59) = 5.00, *p* = .03, partial *η^2^* = .08), fusiform gyrus (F(1,59) = 5.77, *p* = .02, partial *η^2^* = .09), precuneus (F(1,59) = 5.61, *p* = .02, partial *η^2^* = .09), as well as the right lingual (F(1,59) = 10.21, *p* = .002, partial *η^2^* = .15) and postcentral cortices (F(1,59) = 7.11, *p* = .01, partial *η^2^* = .11). Age ∗ group interaction terms did not reach statistical significance for the following regions: volumetric measures of left paracentral cortex and right precuneus, CT in the right frontal pole, fusiform gyrus, precuneus, and lateral occipital cortex, left postcentral cortex, and in bilateral middle frontal regions. For all regions that showed a significant age ∗ group interaction, controls showed a highly significant pattern of decreasing CT with increasing age, which was not observed in 22q11DS participants. Fisher's test for the equivalence of correlations showed that, in four of these regions (left pericalcarine and fusiform regions, right lingual and postcentral cortices), the strength of the correlation was significantly greater in controls relative to 22qDS participants (Supplementary Table 3).

### 3.5 Relationship between MRI indices and positive symptoms in 22q11.2 deletion syndrome

**Fig. 4:**
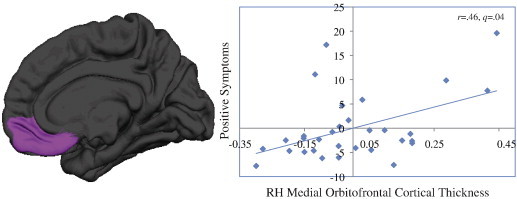
Relationship between increased cortical thickness in the right medial orbitofrontal cortex and positive symptom severity in 22q11DS. Medial orbitofrontal cortical thickness and positive symptoms are residuals, with effects of age and sex regressed out.

Correlations were conducted for each of the CT and SA MRI indices. After FDR correction was applied, there was a significant positive correlation between right medial orbitofrontal CT and positive symptoms in 22q11DS participants (*r* = .46, *q* = .04). Increased CT in the right medial orbitofrontal cortex was associated with increased positive symptoms in 22q11DS (Fig. 4). Additional trend-level associations observed in this analysis are reported in Supplementary Table 4. Of note, trend-level associations were detected between positive symptoms and SA measures in multiple temporal regions: the left (*r* = − .38, *q* = .14) and right middle temporal (*r* = − .34, *q* = .19), right inferior temporal (*r* = − .36, *q* = .15), and temporal pole regions (r = − .34, *q* = .19). In all four regions, as SA decreased, positive symptoms increased in severity in those with 22q11DS.

Correlations were conducted for each of the CT and SA MRI indices. After FDR correction was applied, there was a significant positive correlation between right medial orbitofrontal CT and positive symptoms in 22q11DS participants (*r* = .46, *q* = .04). Increased CT in the right medial orbitofrontal cortex was associated with increased positive symptoms in 22q11DS (Fig. 4). Additional trend-level associations observed in this analysis are reported in Supplementary Table 4. Of note, trend-level associations were detected between positive symptoms and SA measures in multiple temporal regions: the left (*r* = − .38, *q* = .14) and right middle temporal (*r* = − .34, *q* = .19), right inferior temporal (*r* = − .36, *q* = .15), and temporal pole regions (r = − .34, *q* = .19). In all four regions, as SA decreased, positive symptoms increased in severity in those with 22q11DS.

## 4 Discussion

To our knowledge, this is the first study to examine regional differences in brain structures on the basis of their separable components (volume, cortical thickness, and surface area) in 22q11.2 microdeletion syndrome (22q11DS), a recurrent genetic mutation associated with high rates of psychosis. We also investigated age-associated trajectories in these structures in 22q11DS participants vs. typically developing controls, and explored relationships between structural variation in these brain regions and dimensional measures of positive symptom severity. These analyses yielded several novel findings: 1) measures of volume, CT, and SA indicate distinct patterns of group differences, involving reduced cortical volume and SA in occipito-parietal, temporal and anterior cingulate cortices, whereas increased CT was observed in multiple frontal regions and the insula; 2) multiple regions, including the fusiform gyrus, showed cortical thinning with increasing age in typically developing controls, but this pattern of age-associated cortical thinning was not present in 22q11DS; and 3) increased CT in the right medial orbitofrontal cortex was associated with greater severity of positive symptoms in 22q11DS.

### 4.1 Group differences in cortical volume, thickness, and surface area

Analysis of multiple neuroanatomic indices (e.g., volume, CT, and SA) revealed a complex and intriguing pattern of results. Consistent with previous studies, we found volumetric reductions in occipito-parietal (Bearden et al., 2007; Gothelf et al., 2007b; Kates et al., 2001), temporal (Chow et al., 2011; van Amelsvoort et al., 2004) and anterior cingulate cortices (Dufour et al., 2008; Shashi et al., 2010) in 22q11DS; ours is the first study to show that these reductions are driven by decreased SA. Our findings of increased bilateral insular volumes replicate findings from two separate laboratories (Campbell et al., 2006; Simon et al., 2005). Here, we extend upon these findings by showing that increased CT, not changes in SA, drives increased volumes in these regions. Finally, while our findings are in line with prior studies that conclude that cortical volumes of most frontal regions are relatively “intact” in children and adolescents with 22q11DS (Eliez et al., 2000; Kates et al., 2004; Simon et al., 2005), we additionally showed that CT in the bilateral medial orbitofrontal, middle and inferior frontal cortices was significantly increased in those with 22q11DS in comparison to typically developing controls. While the volumetric increase we observed in medial orbitofrontal cortex in 22q11DS was in the opposite direction of the one prior study, to our knowledge, that has reported on orbitofrontal cortical volumes in 22q11DS (Kates et al., 2011b), no other studies have examined indices of CT and SA together in 22q11DS. Our results replicate previous findings using a different methodology in youth ages 9–15 years old, which also found multiple areas of increased CT in 22q11DS relative to healthy controls, primarily in frontal brain regions (Schaer et al., 2009). Interestingly, though CT in the middle frontal region was increased, the volume and SA of this region were both decreased in 22q11DS. It has been shown that SA is more strongly related to volume than is CT (Winkler et al., 2010), providing a possible explanation for findings of increased CT and decreased volume in the same brain region. Again, this finding highlights the importance of examining indices of CT and SA separately.

By examining separate components of volume (i.e., SA and CT) we may begin to better understand the underlying neural dysfunction in 22q11DS, given the distinct neurodevelopmental origins of these two indices (Pontious et al., 2008). Specifically, converging evidence suggests that cortical SA is determined by proliferation of radial unit progenitors, which consist of neuroepithelial cells and radial glial cells (Pontious et al., 2008). Therefore the decreased SA seen in the anterior cingulate and inferior temporal regions in 22q11DS may reflect reduced production of radial unit progenitors in these areas of the cortex. Meanwhile, CT is believed to be determined by intermediate progenitor cells, which produce only neurons and are considered to be transient cells whose job is to amplify cells within the developing cortex (Pontious et al., 2008). Thus, increased cortical thickness, which was seen in multiple frontal regions, may have occurred as a result of over-proliferation of intermediate progenitor cells during corticogenesis. Furthermore, previous studies of 22q11.2 deletion mouse models suggest that reduced dosage of genes within the 1.5-megabase 22q11 minimal critical deleted region results in a disruption of basal progenitor proliferation in the cortex and abnormal migration of interneurons in the medial cortex (Meechan et al., 2009). These findings suggest that disrupted corticogenesis due to basal progenitor defects may play a role in circuit vulnerability and behavioral alterations in the 22q11.2 mouse model (Meechan et al., 2012), which may in turn be relevant to the observed CT and SA alterations in our sample. The findings in the mouse model were restricted to the medial wall and thus may not fully explain the changes in SA and CT observed in our human 22q11DS sample. Nevertheless, Meechan et al.'s intriguing finding of abnormal interneuron migration due to reduced 22q11.2 gene dosage, particularly the Cxcr4 gene, suggests a molecular mechanism for disrupted neurodevelopment in 22q11DS, a possibility which should be investigated further in both animal models and human post-mortem studies. It should also be noted that our findings are constrained by the manner in which FreeSurfer calculates these MRI indices, limiting our neurobiological interpretations. Furthermore, alterations in cortical volume may be linked to other cellular processes, such as decreased dendritic spine density (Glantz and Lewis, 2000), changes in interneurons (Benes et al., 1991), or reduction in neuronal cell size (Pennington et al., 2008). Finally, given that structural MRI is an indirect measure of neuronal processes, our conclusions regarding the underlying neuropathology of the CT and SA differences observed in 22q11DS are necessarily speculative. Nevertheless, it is notable that a recent high-resolution MRI study of the 22q11.2 deletion mouse model (*Df*(*16*)*A*^+/−^) found that the brain abnormalities in these mice recapitulate some of the most common neuroanatomical findings in human patients, particularly increased striatal volume and – consistent with our findings – relative volumetric increases in the frontal lobe in *Df*(*16*)*A*^+/−^ mice compared with wild-type littermates (Ellegood et al., 2013). These similarities highlight the face validity of the 22q11.2 mouse model as a means of advancing our understanding of the critical pathogenic mechanisms involved in abnormal brain development in human 22q11.2 deletion carriers.

Human neuroimaging studies have also shown that SA and CT have separate genetic origins (Panizzon et al., 2009; Winkler et al., 2010). Notably, the genetic dissociation of CT and SA is also supported by animal models, which show that mutation of specific genes involved in neurodevelopment (*PAX6*, *LRP6*, and *NGN1*/*2*) affects the number of progenitor cells, resulting in concomitant increases in CT but not SA, a finding which was validated in humans (Baala et al., 2007; Glaser et al., 1994; Joyner et al., 2009). In contrast, variation in *MECP2*, a gene known to be involved in brain structural development, was associated with variation in SA but not CT within specific cortical regions (e.g., cuneus, fusiform, inferior frontal gyrus; Joyner et al., 2009). Thus, the differential patterns of brain disruption that we observed (e.g., increased CT in frontal and insular cortices, but decreased SA in the anterior cingulate and inferior temporal regions) suggest that these alterations may also reflect disruptions of different genetic mechanisms in 22q11DS. In addition, evidence suggests that particular genes within the 22q11.2 region (e.g., *RANBP1*, *CADC45l*, *Cxcr4*) may contribute to the observed alterations in cortical development in the mouse (Meechan et al., 2009, 2012). Future studies examining the relationship between these genes and cortical development, in human and animal models, are necessary to expand upon and validate these findings.

### 4.2 Relationships between MRI indices and positive symptoms in 22q11.2 deletion syndrome

Importantly, we found that increased CT in the right medial orbitofrontal cortex, a brain region critically involved in social cognition (Amodio and Frith, 2006), was associated with more severe positive symptoms in 22q11DS. Though increased orbitofrontal thickness is not typically reported in individuals with idiopathic schizophrenia, there is substantial heterogeneity in findings, with some studies finding increased orbitofrontal cortical volume in first-episode schizophrenia (Szeszko et al., 1999), while others have found reduced volumes (Convit et al., 2001) (Gur et al., 2000) or no significant differences (Sapara et al., 2007). These disparate findings may be due to the heterogeneous nature of schizophrenia, thus highlighting the value of investigating well characterized, homogenous subtypes of schizophrenia. However, our findings provide preliminary evidence that neuroanatomic regions that are particularly relevant for socially oriented interactions (Amodio and Frith, 2006) also play a role in the development of psychosis in 22q11DS. Our recent behavioral studies show that social cognition performance is a better predictor of positive symptoms in 22q11DS than non-social cognitive measures, supporting this notion (Jalbrzikowski et al., 2012).

We also found several trend-level relationships, including reduced SA in multiple temporal regions associated with greater severity of positive symptoms in 22q11DS. These findings are consistent with prior cross-sectional studies of both adults (Chow et al., 2002, 2011) and children with 22q11DS (Bearden et al., 2004), and additionally suggest that SA reductions may be driving the previously observed associations between temporal lobe volume and symptom severity. These findings also support existing longitudinal studies of both clinical high-risk youth and adolescents with 22q11DS, which have shown that volumetric decreases in temporal regions are associated with the development of more severe prodromal or psychotic symptomatology (Kates et al., 2011a; Takahashi et al., 2009). Taken together, these findings provide corroborating evidence that aberrant temporal lobe morphology may play a critical role in the development of psychosis in both 22q11DS and the broader population.

The additional trend-level associations we observed between reduced SA in occipital regions (e.g., pericalcarine, lingual) and increased positive symptoms have, to our knowledge, not been reported in previous studies; however, this may be due to the fact that most studies to date have not taken an unbiased approach to examine brain–behavior relationships across all cortical regions.

### 4.3 Age-associated changes in cortical thickness

We also found several age ∗ group interactions in occipital (i.e., pericalcarine, fusiform, and lingual regions) and parietal regions (i.e., precuneus, postcentral regions), in which typically developing controls showed highly significant cortical thinning with increasing age, as found in previous studies of typically developing adolescents and young adults (Tamnes et al., 2010). In contrast, this pattern was not observed in 22q11DS individuals. These findings suggest that adolescent cortical maturation in parieto-occipital regions may be disrupted in 22q11DS. These findings, however, need to be confirmed with prospective longitudinal studies and larger sample sizes.

Furthermore, two of these regions, the fusiform gyrus and precuneus, are known to be important for socially relevant processes (Cavanna and Trimble, 2006; Schultz et al., 2003). Thus, disruption of cortical maturation in these areas may be related to social cognitive impairments observed in youth with 22q11DS (Campbell et al., 2011; Ho et al., 2012). Thus, in the future, it will be important to examine whether structural and functional variations in socially-relevant brain regions are related to social cognitive development in 22q11DS.

### 4.4 Study limitations

Several limitations to this study should be noted. First, given the cross-sectional design we were unable to investigate baseline neuroanatomic measures, or change in these variables over time, as predictors of subsequent psychotic symptom development. Given that recent findings suggest that regional neuroanatomic changes over time predict prodromal symptoms, assessed by the SIPS (Kates et al., 2011a), it is critical that future studies incorporate a longitudinal approach. Additionally, it will be important to investigate whether our cross-sectional finding suggesting a disrupted trajectory of CT in the middle frontal region in 22q11DS is replicated in a within-subject, longitudinal design. Also, regarding the association of our neuroanatomic measures with positive symptoms, the majority of the 22q11DS participants in our sample were not fully psychotic; only three 22q11DS participants had a diagnosis of a psychotic disorder, and thus we used dimensional measures of positive symptoms as our dependent variable. Nevertheless, given that psychotic symptoms are continuously distributed in the general population (Ahmed et al., 2011), utilizing a dimensional approach may be more powerful than investigating psychotic symptoms as a categorical variable.

### 4.5 Implications for idiopathic schizophrenia

Given that 22q11DS is one of the greatest known risk factors for psychosis, it is important to examine how these results relate to the existing literature on idiopathic schizophrenia. At first glance, the results may seem counter-intuitive, given that many of our results were driven by increased CT, while exaggerated cortical thinning in multiple brain regions has been associated with conversion to psychosis (Sun et al., 2009; Takahashi et al., 2009), along with poorer neuropsychological functioning and more severe clinical symptomatology in patients with established schizophrenia (Cobia et al., 2012). While the direction of effects observed in frontal regions in our 22q11DS sample may not have been predicted based on existing literature on idiopathic psychosis, it may be that there are multiple pathways of disruption in particular brain regions relevant for the development of psychosis, resulting in similar downstream phenotypic effects. While many researchers speculate that increased cortical thinning in idiopathic schizophrenia is suggestive of an overly aggressive synaptic pruning process (Hoffman and McGlashan, 1997; Keshavan et al., 1994), it is possible that the increased CT observed in primarily frontal regions in 22q11DS may be due to neuronal over-proliferation in these brain regions, resulting in a similar behavioral phenotype (e.g., positive symptoms). Use of an unbiased, whole-brain analytic approach revealed additional neuroanatomic differences in 22q11DS individuals that are unique to this population and not characteristic of idiopathic schizophrenia (i.e., occipito-parietal regions, Bearden et al., 2007; Gothelf et al., 2007b; Kates et al., 2001). Therefore, the overall neuroanatomic profile of 22q11DS individuals may represent an independent manifestation of aberrant brain development. However, given the clear and consistent association of the 22q11.2 deletion with schizophrenia risk (Debbane et al., 2006; Gothelf et al., 2007a; Schneider et al., 2013), future investigations that focus on cortical regions directly related to psychotic symptomatology in 22q11DS (e.g., medial frontal regions) may be particularly informative regarding pathophysiologic mechanisms relevant to psychosis risk and protective factors in this population.

### 4.6 Conclusions

Findings from the current study suggest that neuroanatomic abnormalities in brain regions disproportionately affected in idiopathic schizophrenia may be driven by distinct neurodevelopmental processes; as such, different mechanisms may underlie disruptions associated with psychosis risk, both in the context of 22q11.2 microdeletion syndrome and the general population. Future experiments in animal models of the 22q11.2 deletion, in which specific genes in the region are rescued, may provide direct links between mechanisms of disruption in cortical development and psychosis pathogenesis.

The following are the Supplementary data related to this article.

Supplementary text Description of manual edits carried out in FreeSurfer.

Supplementary Table 1 Comparisons of cortical volume, cortical thickness and surface area across scanner locations.

Supplementary Table 2 Global neuroanatomic measures in 22q11DS vs. controls.

Supplementary Table 3 Fisher r-to-z transformation results for significant age ∗ group interactions between cortical regions in 22q11DS vs. controls.

Supplementary Table 4 Significant and trend-level associations between cortical regions and positive symptoms in 22q11DS participants.

Supplementary data to this article can be found online at http://dx.doi.org/10.1016/j.nicl.2013.09.013.

## Figures and Tables

**Table 1:** Demographic and clinical characteristics of study participants.

	22q11DS participants (n = 31)	Healthy comparison participants (n = 34)	
Age (years ± SD)	16.4 (4.2)	15.7 (3.8)	*p* = .47
Participant education (years ± SD)	8.5 (3.7)	9.2 (3.6)	*p* = .49
Parental education (years ± SD)	16.2 (2.9)	15.4 (2.9)	*p* = .27
Gender (n, % female)	16 (52%)	14 (42%)	*p* = .39
Race (Asian/African American/Caucasian/multiple)	0/1/27/3	3/4/22/5	*p* = .13
Ethnicity (n, % Latino)	7 (23%)	13 (38%)	*p* = .17
Psychotic disorder diagnosis (n, %)	3 (10%)	NA	
SIPS positive symptoms (mean ± SD)	7.1 (6.9)	1.1 (1.6)	*p* < .001
SIPS negative symptoms	9.2 (6.4)	1.1 (1.7)	*p* < .001
SIPS disorganized symptoms	4.7 (3.7)	0.7 (1.0)	*p* < .001
SIPS general symptoms	4.9 (5.0)	1.1 (1.4)	*p* = .001
Psychotic symptoms (positive + negative symptoms on SIPS)	16.3 (11.9)	2.2 (3.0)	*p* < .001
Psychotropic medication (n, none/antipsychotics/antidepressants)	22/3/6	NA	

**Table 2:** Group comparisons for neuroanatomic measures: 22q11DS participants versus typically developing controls. Bold values indicate a statistically significant difference between neuroanatomic measures in 22q11DS versus controls.

		Volume			Cortical thickness			Surface area		
Region of interest	Hemisphere	F	FDR *q*-value	↑ or ↓ in 22q11DS	F	FDR *q*-value	↑ or ↓ in 22q11DS	F	FDR *q*-value	↑ or ↓ in 22q11DS
Inferior frontal	L	0.19	0.87		**18**.**68**	**0**.**0008**	**↑**	0.21	0.86	
	R	0.00	0.98		6.88^a^	0.06	↑	0.71	0.70	
Middle frontal	L	4.71^a^	0.13	↓	**18**.**79**	**0**.**0008**	**↑**	**15**.**24**	**0**.**003**	**↓**
	R	**13**.**96**	**0**.**003**	**↓**	**25**.**94**	**0**.**0002**	**↑**	**41**.**09**	**0**.**00002**	**↓**
Superior frontal	L	1.07	0.62		1.98	0.41		4.01^a^	0.16	↓
	R	0.38	0.79		4.33^a^	0.15	↑	0.05	0.93	
Medial orbitofrontal	L	6.77^a^	0.06	↑	**14**.**21**	**0**.**004**	**↑**	0.01	0.98	
	R	**14**.**68**	**0**.**003**	**↑**	**18**.**70**	**0**.**0008**	**↑**	0.63	0.71	
Lateral orbitofrontal	L	2.52	0.32		5.19^a^	0.09	↑	0.03	0.94	
	R	0.87	0.68		4.30^a^	0.15	↑	0.27	0.84	
Frontal pole	L	0.624	0.71		**16**.**18**	**0**.**002**	**↑**	0.341	0.75	
	R	1.74	0.46		0.04	0.94		1.38	0.46	
Insula	L	**13**.**01**	**0**.**005**	**↑**	**19**.**86**	**0**.**0007**	**↑**	4.02^a^	0.16	↑
	R	**22**.**20**	**0**.**0004**	**↑**	**19**.**48**	**0**.**0008**	**↑**	3.24	0.23	
Anterior cingulate	L	**12**.**43**	**0**.**007**	**↓**	0.83	0.68		**16**.**80**	**0**.**002**	**↓**
	R	**9**.**27**	**0**.**02**	**↓**	0.42	0.78		**16**.**59**	**0**.**002**	**↓**
Isthmus cingulate	L	0.00	0.97		3.16	0.23		1.37	0.53	
	R	0.54	0.69		0.32	0.81		0.816	0.68	
Posterior cingulate	L	3.15	0.23		0.03	0.94		5.26^a^	0.11	↓
	R	0.66	0.71		0.37	0.79		0.92	0.67	
Precentral	L	0.75	0.69		2.10	0.38		0.378	0.79	
	R	**7**.**82**	**0**.**04**	**↓**	5.48^a^	0.10	↑	1.65	0.48	
Paracentral	L	0.14	0.90		**11**.**33**	**0**.**007**	**↑**	6.13^a^	0.07	↓
	R	0.02	0.96		**8**.**96**	**0**.**024**	**↑**	1.46	0.52	
Postcentral	L	0.04	0.94		6.47^a^	0.07	↑	6.91^a^	0.06	↓
	R	2.75	0.29		1.82	0.44		**10**.**8**	**0**.**01**	**↓**
Superior parietal	L	**18**.**68**	**0**.**0008**	**↓**	0.19	0.87		**22**.**6**	**0**.**00034**	**↓**
	R	**15**.**65**	**0**.**002**	**↓**	1.78	0.45		**28**.**07**	**0**.**00009**	**↓**
Supramarginal	L	0.801	0.68		**7**.**17**	0.05	**↑**	0.1	0.91	
	R	0.628	0.71		**11**.**29**	**0**.**007**	**↑**	0.016	0.96	
Inferior parietal	L	0.11	0.91		2.85	0.27		0.89	0.68	
	R	0.07	0.92		4.09^a^	0.16	↑	1.53	0.50	
Lateral occipital	L	1.9	0.42		0.37	0.79		3.77^a^	0.18	↓
	R	1.53	0.50		0.47	0.78		4.40^a^	0.15	↓
Precuneus	L	**8**.**838**	**0**.**02**	**↓**	4.01^a^	0.16	↑	**28**.**87**	**0**.**00005**	**↓**
	R	4.02^a^	0.16	↓	2.89	0.27		**16**.**66**	**0**.**002**	**↓**
Cuneus	L	**39**.**11**	**0**.**00005**	**↓**	3.61^a^	0.19	↑	**83**.**39**	**0**.**000001**	**↓**
	R	**20**.**09**	**0**.**0007**	**↓**	0.433	0.78		**39**.**93**	**0**.**00001**	**↓**
Pericalcarine	L	2.28	0.36		**11**.**53**	**0**.**007**	**↑**	**16**.**04**	**0**.**002**	**↓**
	R	3.65^a^	0.19	↓	4.93^a^	0.12	↑	**13**.**83**	**0**.**004**	**↓**
Lingual	L	6.51^a^	0.06	↓	7.08^a^	0.05	↑	**20**.**65**	**0**.**0006**	**↓**
	R	5.69^a^	0.09	↓	**9**.**58**	**0**.**02**	**↑**	**21**.**26**	**0**.**0005**	**↓**
Fusiform	L	**9**.**53**	**0**.**02**	**↓**	0.11	0.91		**12**.**91**	**0**.**007**	**↓**
	R	**8**.**45**	**0**.**03**	**↓**	5.63^a^	0.09	↑	**24**.**55**	**0**.**0002**	**↓**
Inferior temporal	L	**23**.**07**	**0**.**0003**	**↓**	0.17	0.87		**38**.**19**	**0**.**00002**	**↓**
	R	**9**.**56**	**0**.**02**	**↓**	0.80	0.68		**15**.**59**	**0**.**002**	**↓**
Middle temporal	L	0.36	0.79		2.36	0.35		**9**.**59**	**0**.**02**	**↓**
	R	0.06	0.92		**7**.**66**	**0**.**04**	**↑**	4.00^a^	0.16	↓
Superior temporal	L	2.14	0.38		4.85^a^	0.13	↓	0.01	0.96	
	R	5.30^a^	0.11	↓	0.00	0.98		6.64^a^	0.06	↓
Banks of superior temporal sulcus	L	0.79	0.68		0.73	0.69		1.61	0.48	
	R	6.38^a^	0.07	↓	0.44	0.78		**11**.**51**	**0**.**007**	**↓**
Transverse temporal pole	L	0.461	0.78		0.33	0.81		1.55	0.50	
	R	4.7^a^	0.13	↓	0.98	0.65		4.33^a^	0.15	↓
Temporal pole	L	1.41	0.53		3.31	0.22		**18**.**96**	**0**.**0008**	**↓**
	R	3.39^a^	0.21	↓	1.04	0.63		6.88^a^	0.06	↓
Entorhinal	L	0.07	0.92		0.115	0.91		3.545	0.31	
	R	0.03	0.94		2.75	0.29		0.082	0.92	
Parahippocampal	L	4.10^a^	0.16	↓	**9**.**45**	**0**.**02**	**↓**	0.00	0.98	
	R	0.23	0.85		0.18	0.87		0.61	0.71	

a Indicates measures that showed statistical trends in the results (*q*-values > .05 and < .20).
