# Supplementary material for: Structural abnormalities in cortical volume, thickness, and surface area in 22q11.2 microdeletion syndrome: Relationship with psychotic symptoms
Source: Neuroimage Clin. 2013 Oct 14;3:405–15. doi: 10.1016/j.nicl.2013.09.013 (PMC3814944; doi:10.1016/j.nicl.2013.09.013)
Supplement: Supplementary text — Description of manual edits carried out in FreeSurfer. [file mmc1.doc]

**Supplementary Text: Description of manual edits carried out in Freesurfer**

Manual edits were carried out as follows:

1. Recon-all was run on each subject.
2. For each subject, the orig.mgz file was examined in tkmedit for ringing, increased signal-to-noise ratio, or inhomogeneity. The scan was also examined to ensure that full coverage was acquired during the scan.
3. Each scan was manually examined to make sure that intensity normalization and the Talaraich transformation were performed successfully.
4. If necessary, pial edits were then made in brainmask.mgz for each scan in tkmedit. Areas of focus included: removal of dura matter, cerebellum, tentorium cerebelli, and/or optic nerve. If these areas were misclassified as gray matter, then pial edits were made to remove these areas from the gray matter classification.
5. If necessary, white matter and control point edits were conducted in tkmedit. White matter and control points were used if white matter regions were not appropriately specified as white matter.
6. The scan was re-submitted through the appropriate point in recon-all (i.e., recon-all autorecon2-cp –autorecon3)
7. Final quality assurance was then conducted on the scan in Freeview. The inflated and pial views were examined for any errors. Cortical parcellations were also viewed to ensure accuracy.
