# Supplementary material for: Structural abnormalities in cortical volume, thickness, and surface area in 22q11.2 microdeletion syndrome: Relationship with psychotic symptoms
Source: Neuroimage Clin. 2013 Oct 14;3:405–15. doi: 10.1016/j.nicl.2013.09.013 (PMC3814944; doi:10.1016/j.nicl.2013.09.013)
Supplement: Supplementary Table 1 — Comparisons of cortical volume, cortical thickness and surface area across scanner locations. [file mmc2.doc]

Supplementary Table 1: Comparisons of cortical volume, cortical thickness and surface area across scanner locations.

|  |  | Volume | | | | Cortical Thickness | | | Surface Area | | |
| --- | --- | --- | --- | --- | --- | --- | --- | --- | --- | --- | --- |
| Region of Interest | Hemi-sphere | F | *p*-value | FDR *q*-value | F | | *p*-value | FDR *q*-value | F | *p*-value | FDR *q*-value |
| Inferior Frontal | L | 0.01 | 0.94 | 0.99 | 0.87 | | 0.35 | 0.99 | 0.25 | 0.62 | 0.99 |
|  | R | 0.01 | 0.93 | 0.99 | 0.12 | | 0.73 | 0.99 | 0.07 | 0.79 | 0.99 |
| Middle Frontal | L | 1.53 | 0.22 | 0.99 | 0.22 | | 0.64 | 0.99 | 0.95 | 0.34 | 0.99 |
|  | R | 0.56 | 0.46 | 0.99 | 0.02 | | 0.88 | 0.99 | 0.33 | 0.57 | 0.99 |
| Superior Frontal | L | 0.14 | 0.71 | 0.99 | 0.09 | | 0.76 | 0.99 | 0.22 | 0.64 | 0.99 |
|  | R | 0.30 | 0.59 | 0.99 | 0.00 | | 0.98 | 0.99 | 0.29 | 0.60 | 0.99 |
| Medial Orbitofrontal | L | 1.61 | 0.21 | 0.99 | 0.07 | | 0.79 | 0.99 | 2.39 | 0.13 | 0.99 |
|  | R | 0.06 | 0.81 | 0.99 | 0.04 | | 0.85 | 0.99 | 0.00 | 0.98 | 0.99 |
| Lateral Orbitofrontal | L | 2.45 | 0.12 | 0.99 | 0.14 | | 0.71 | 0.99 | 2.57 | 0.11 | 0.99 |
|  | R | 1.91 | 0.17 | 0.99 | 0.95 | | 0.33 | 0.99 | 1.48 | 0.23 | 0.99 |
| Frontal Pole | L | 5.12 | 0.03 | 0.90 | 0.42 | | 0.52 | 0.99 | 3.72 | 0.06 | 0.98 |
|  | R | 1.18 | 0.28 | 0.99 | 1.97 | | 0.17 | 0.99 | 0.18 | 0.68 | 0.99 |
| Insula | L | 0.01 | 0.98 | 0.99 | 1.04 | | 0.31 | 0.99 | 0.19 | 0.67 | 0.99 |
|  | R | 0.12 | 0.73 | 0.99 | 0.02 | | 0.90 | 0.99 | 0.17 | 0.68 | 0.99 |
| Anterior Cingulate | L | 0.30 | 0.58 | 0.99 | 1.30 | | 0.26 | 0.99 | 0.00 | 0.99 | 0.99 |
|  | R | 0.43 | 0.52 | 0.99 | 0.11 | | 0.74 | 0.99 | 0.03 | 0.87 | 0.99 |
| Isthmus Cingulate | L | 0.01 | 0.93 | 0.99 | 0.22 | | 0.64 | 0.99 | 0.00 | 0.98 | 0.99 |
|  | R | 0.95 | 0.34 | 0.99 | 0.86 | | 0.36 | 0.99 | 1.50 | 0.23 | 0.99 |
| Posterior Cingulate | L | 8.76 | 0.004 | 0.36 | 0.00 | | 0.96 | 0.99 | 11.01 | 0.001 | 0.18 |
|  | R | 4.88 | 0.03 | 0.9 | 0.09 | | 0.76 | 0.99 | 5.67 | 0.02 | 0.9 |
| Precentral | L | 0.00 | 0.98 | 0.99 | 0.00 | | 0.96 | 0.99 | 0.03 | 0.86 | 0.99 |
|  | R | 0.09 | 0.77 | 0.99 | 0.44 | | 0.51 | 0.99 | 0.68 | 0.41 | 0.99 |
| Paracentral | L | 1.74 | 0.19 | 0.99 | 1.91 | | 0.17 | 0.99 | 1.08 | 0.30 | 0.99 |
|  | R | 0.61 | 0.44 | 0.99 | 0.40 | | 0.53 | 0.99 | 0.11 | 0.74 | 0.99 |
| Postcentral | L | 0.75 | 0.39 | 0.99 | 3.70 | | 0.06 | 0.9 | 0.04 | 0.85 | 0.99 |
|  | R | 0.46 | 0.50 | 0.99 | 0.33 | | 0.57 | 0.99 | 0.06 | 0.80 | 0.99 |
| Superior Parietal | L | 0.16 | 0.69 | 0.99 | 0.01 | | 0.93 | 0.99 | 0.07 | 0.79 | 0.99 |
|  | R | 0.06 | 0.81 | 0.99 | 0.13 | | 0.72 | 0.99 | 0.01 | 0.93 | 0.99 |
| Supramarginal | L | 0.56 | 0.46 | 0.99 | 2.96 | | 0.09 | 0.99 | 1.97 | 0.17 | 0.99 |
|  | R | 1.00 | 0.32 | 0.99 | 0.56 | | 0.46 | 0.99 | 1.32 | 0.25 | 0.99 |
| Inferior Parietal | L | 0.07 | 0.80 | 0.99 | 0.14 | | 0.71 | 0.99 | 0.14 | 0.71 | 0.99 |
|  | R | 1.80 | 0.19 | 0.99 | 0.76 | | 0.39 | 0.99 | 0.99 | 0.32 | 0.99 |
| Lateral Occipital | L | 0.16 | 0.69 | 0.99 | 3.88 | | 0.05 | 0.98 | 0.02 | 0.89 | 0.99 |
|  | R | 0.41 | 0.52 | 0.99 | 0.15 | | 0.70 | 0.99 | 0.39 | 0.54 | 0.99 |
| Precuneus | L | 0.42 | 0.52 | 0.99 | 0.04 | | 0.83 | 0.99 | 0.94 | 0.34 | 0.99 |
|  | R | 0.13 | 0.73 | 0.99 | 0.49 | | 0.49 | 0.99 | 0.79 | 0.38 | 0.99 |
| Cuneus | L | 0.14 | 0.71 | 0.99 | 0.21 | | 0.66 | 0.99 | 0.08 | 0.77 | 0.99 |
|  | R | 0.45 | 0.51 | 0.99 | 0.01 | | 0.93 | 0.99 | 0.63 | 0.43 | 0.99 |
| Pericalcarine | L | 0.03 | 0.87 | 0.99 | 0.63 | | 0.43 | 0.99 | 0.03 | 0.87 | 0.99 |
|  | R | 0.12 | 0.73 | 0.99 | 0.02 | | 0.88 | 0.99 | 0.14 | 0.71 | 0.99 |
| Lingual | L | 0.08 | 0.77 | 0.99 | 2.00 | | 0.16 | 0.99 | 0.01 | 0.94 | 0.99 |
|  | R | 0.26 | 0.61 | 0.99 | 0.27 | | 0.61 | 0.99 | 0.03 | 0.86 | 0.99 |
| Fusiform | L | 0.42 | 0.52 | 0.99 | 0.04 | | 0.85 | 0.99 | 0.16 | 0.69 | 0.99 |
|  | R | 0.45 | 0.50 | 0.99 | 0.07 | | 0.79 | 0.99 | 0.05 | 0.82 | 0.99 |
| Inferior Temporal | L | 1.83 | 0.18 | 0.99 | 3.09 | | 0.09 | 0.99 | 0.43 | 0.51 | 0.99 |
|  | R | 0.87 | 0.36 | 0.99 | 2.79 | | 0.10 | 0.99 | 1.39 | 0.24 | 0.99 |
| Middle Temporal | L | 0.003 | 0.96 | 0.99 | 0.13 | | 0.72 | 0.99 | 0.05 | 0.83 | 0.99 |
|  | R | 0.14 | 0.71 | 0.99 | 6.78 | | 0.01 | 0.6 | 0.27 | 0.61 | 0.99 |
| Superior Temporal | L | 0.13 | 0.72 | 0.99 | 0.72 | | 0.40 | 0.99 | 0.00 | 0.99 | 0.99 |
|  | R | 0.20 | 0.65 | 0.99 | 0.14 | | 0.71 | 0.99 | 0.01 | 0.92 | 0.99 |
| Banks of Superior Temporal Sulcus | L | 0.29 | 0.59 | 0.99 | 0.32 | | 0.57 | 0.99 | 0.51 | 0.48 | 0.99 |
|  | R | 2.99 | 0.09 | 0.99 | 0.96 | | 0.33 | 0.99 | 1.85 | 0.18 | 0.99 |
| Transverse Temporal Pole | L | 0.36 | 0.55 | 0.99 | 0.00 | | 0.98 | 0.99 | 0.03 | 0.87 | 0.99 |
|  | R | 1.2 | 0.28 | 0.99 | 1.76 | | 0.19 | 0.99 | 0.10 | 0.75 | 0.99 |
| Temporal Pole | L | 4.02 | 0.05 | 0.98 | 0.52 | | 0.48 | 0.99 | 2.53 | 0.12 | 0.99 |
|  | R | 0.30 | 0.59 | 0.99 | 1.95 | | 0.17 | 0.99 | 2.35 | 0.13 | 0.99 |
| Entorhinal | L | 0.29 | 0.59 | 0.99 | 0.58 | | 0.45 | 0.99 | 0.79 | 0.38 | 0.99 |
|  | R | 0.01 | 0.94 | 0.99 | 0.39 | | 0.53 | 0.99 | 0.10 | 0.75 | 0.99 |
| Parahippocampal | L | 1.38 | 0.25 | 0.99 | 1.39 | | 0.24 | 0.99 | 0.20 | 0.89 | 0.99 |
|  | R | 4.06 | 0.05 | 0.98 | 0.31 | | 0.58 | 0.99 | 1.44 | 0.24 | 0.99 |
