# Supplementary material for: Structural abnormalities in cortical volume, thickness, and surface area in 22q11.2 microdeletion syndrome: Relationship with psychotic symptoms
Source: Neuroimage Clin. 2013 Oct 14;3:405–15. doi: 10.1016/j.nicl.2013.09.013 (PMC3814944; doi:10.1016/j.nicl.2013.09.013)
Supplement: Supplementary Table 2 — Global neuroanatomic measures in 22q11DS vs. controls. [file mmc3.doc]

Supplementary Table 2: Global neuroanatomic measures in 22q11DS vs. controls.

|  | 22q11DS (N=31) | Controls (N=34) | Univariate ANOVA | |
| --- | --- | --- | --- | --- |
|  | Mean  (SD) | Mean  (SD) | F | *p*-value |
| Total Intracranial Volume (mm^3^) | 1395304.36 (197817.77) | 1481054.96  (149288.18) | 4.62 | .036 |
| Total Cortical Volume (mm^3^) | 492233.87  (59788.90) | 538515.27  (58924.62) | 9.44 | .003 |
| Total Cortical Surface Area (mm^2^) | 156421.42 (19201.32) | 175781.06  (15543.86) | 23.58 | 0.000009 |
| Overall Mean Cortical Thickness (mm) | 2.71  (0.09) | 2.65  (0.10) | 13.75 | 0.000458 |
