# Supplementary material for: Structural abnormalities in cortical volume, thickness, and surface area in 22q11.2 microdeletion syndrome: Relationship with psychotic symptoms
Source: Neuroimage Clin. 2013 Oct 14;3:405–15. doi: 10.1016/j.nicl.2013.09.013 (PMC3814944; doi:10.1016/j.nicl.2013.09.013)
Supplement: Supplementary Table 3 — Fisher r-to-z transformation results for significant age ∗ group interactions between cortical regions in 22q11DS vs. controls. [file mmc4.doc]

Supplementary Table 3: Fisher r-to-z transformation results for significant age*group interactions between cortical regions in 22q11DS vs. controls.

|  | Controls (N=34) | | 22q11DS(N=31) | | Z-score | *p-*value |
| --- | --- | --- | --- | --- | --- | --- |
| Cortical Region | *r* | *p* | *r* | *p* |  |  |
| LH pericalcarine | -.53, | .001 | -.03 | .88 | 2.15 | 0.03 |
| LH fusiform | -.49, | .003 | .05 | .79 | 2.25 | 0.02 |
| LH precuneus | -.69 | .000005 | -.43 | .02 | 1.49 | 0.13 |
| RH lingual | -.66 | .00002 | -.05 | .81 | 2.85 | .004 |
| RH postcentral | -.59 | .0002 | -.11 | .56 | 2.18 | .029 |
