# Supplementary material for: Structural abnormalities in cortical volume, thickness, and surface area in 22q11.2 microdeletion syndrome: Relationship with psychotic symptoms
Source: Neuroimage Clin. 2013 Oct 14;3:405–15. doi: 10.1016/j.nicl.2013.09.013 (PMC3814944; doi:10.1016/j.nicl.2013.09.013)
Supplement: Supplementary Table 4 — Significant and trend-level associations between cortical regions and positive symptoms in 22q11DS participants. [file mmc5.doc]

Supplementary Table 4: Significant and trend-level associations between cortical regions and positive symptoms in 22q11DS participants.

| Region | Cortical measurement | *r* | *q-*value |
| --- | --- | --- | --- |
| **RH medial orbitofrontal** | **thickness** | **.46** | **.04** |
| LH posterior cingulate | thickness | .39 | .12 |
| RH pericalcarine | thickness | -.38 | .14 |
| RH precentral | thickness | -.35 | .16 |
| RH lingual | surface area | -.35 | .16 |
| LH middle temporal | surface area | -.38 | .14 |
| RH middle temporal | surface area | -.34 | .19 |
| RH inferior temporal | surface area | -.36 | .15 |
| RH temporal pole | surface area | -.34 | .19 |
